# Laparoscopic sacrocolpopexy compared with open abdominal sacrocolpopexy for vault prolapse repair: a randomised controlled trial

**DOI:** 10.1007/s00192-017-3296-5

**Published:** 2017-04-17

**Authors:** Anne-Lotte W. M. Coolen, Anique M. J. van Oudheusden, Ben Willem J. Mol, Hugo W. F. van Eijndhoven, Jan-Paul W. R. Roovers, Marlies Y. Bongers

**Affiliations:** 1 Department of Gynaecology and Obstetrics, Máxima Medical Centre, De Run 4600, 5500 MB Veldhoven, The Netherlands; 2 Department of Gynaecology and Obstetrics, The Robinson Research Institute, School of Paediatrics and Reproductive Health, University of Adelaide, 5000 Adelaide, SA Australia; 3 Department of Gynaecology and Obstetrics, The South Australian Health and Medical Research Institute, Adelaide, Australia; 4 Department of Gynaecology and Obstetrics, Isala Klinieken, Dokter van Heesweg 2, 8025 AB Zwolle, The Netherlands; 5 Department of Gynaecology and Obstetrics, Academic Medical Centre Amsterdam, Meibergdreef 9, 1105 AZ Amsterdam, The Netherlands; 6 Department of Gynaecology and Obstetrics, University of Maastricht, Minderbroedersberg 4–6, 6211 LK Maastricht, The Netherlands

**Keywords:** Pelvic organ prolapse, Sacral colpopexy, Sacrocolpopexy, Vault prolapse

## Abstract

**Introduction and hypothesis:**

The objective was to evaluate the functional outcome after laparoscopic sacrocolpopexy versus open sacrocolpopexy in women with vault prolapse.

**Methods:**

A multicentre randomised controlled trial was carried out at four teaching and two university hospitals in the Netherlands in women with symptomatic vault prolapse requiring surgical treatment. Participants were randomised for laparoscopic or open sacrocolpopexy. Primary outcome was disease-specific quality of life measured using the Urinary Distress Inventory (UDI) questionnaire at 12 months’ follow-up. Secondary outcomes included anatomical outcome and perioperative data. We needed 74 participants to show a difference of 10 points on the prolapse domain of the UDI 12 months after surgery (power of 80%, α error 0.05).

**Results:**

Between 2007 and 2012, a total of 74 women were randomised. Follow-up after 12 months showed no significant differences in domain scores of the UDI between the two groups. After 12 months, both groups reported a UDI score of 0.0 (IQR: 0–0) for the domain “genital prolapse”, which was the primary outcome. There were no significant differences between the two groups (*p* = 0.93). The number of severe complications was 4 in the laparoscopic group versus 7 in the open abdominal group (RR 0.57; 95% CI 0.50–2.27). There was less blood loss and a shorter hospital stay after laparoscopy; 2 (IQR 2–3) versus 4 (IQR 3–5) days, which was statistically different. There was no significant difference in anatomical outcome at 12 months.

**Conclusion:**

Our trial provides evidence to support a laparoscopic approach when performing sacrocolpopexy, as there was less blood loss and hospital stay was shorter, whereas functional and anatomical outcome were not statistically different.

## Introduction

Post-hysterectomy vaginal vault prolapse has a reported incidence of 0.36 to 3.6 per 1,000 woman years or a cumulative incidence of 0.5% [1, 2]. Abdominal sacrocolpopexy (ASC) is the most effective treatment for vaginal vault prolapse, with a success rate of 93–99%, and is now considered the first-choice treatment for vaginal vault prolapse [3–8]. Sacrocolpopexy is a procedure designed to treat apical compartment prolapse, including uterine or vaginal vault prolapse, in addition to multi-compartment prolapse [9, 10].

According to a Cochrane review on the subject, ASC led to a lower rate of recurrent vault prolapse and dyspareunia compared with vaginal sacrospinous ligament fixation [3]. Nevertheless, ASC is also associated with a longer operative time, recovery period and higher cost [11].

Laparoscopic sacrocolpopexy was first reported in 1994 [12]. Since then, it has gained in popularity, before any clinical advantage over the open abdominal procedure was proven. Although the literature regarding laparoscopic sacrocolpopexy was limited and prospective comparative randomised trials were lacking, the laparoscopic sacrocolpopexy has been widely adopted by pelvic reconstructive surgeons. Laparoscopic sacrocolpopexy has potential advantages over laparotomy, as morbidity, hospital stay, postoperative pain and recovery are all supposed to be less. Moreover, the aesthetic result is better after minimally invasive sacrocolpopexy. However, the laparoscopic approach is more challenging and the literature reports a long learning curve associated with this technique [13, 14]. More importantly, it is unknown if the laparoscopic mesh fixation to the promontory results in an equal anatomical outcome, as it has been stated that as part of the laparoscopic approach, the fixation point is higher, which could result in a more vertical position of the vagina.

Previous studies comparing LSC with ASC showed less blood loss and a significantly shorter hospital stay in the laparoscopic group [15–17]. A randomised controlled trial comparing open laparoscopic with abdominal sacrocolpopexy in patients with a symptomatic vault prolapse, which was published during the follow-up period of our trial, reported significantly less blood loss, a higher haemoglobin level and a shorter hospital stay in favour of the laparoscopic group. There was no significant difference in anatomical outcome between the two groups [15]. The exclusion criteria of the published study were very strict, and only patients with at least a grade 2 vault prolapse, a BMI less than 35 and without urinary stress incontinence were included [15]. This does not match the patient population of the general practice. Our trial creates a realistic reflection of daily practice.

Considering the lack of evidence, we performed a randomised trial comparing LSC with ASC using disease-specific quality of life as the primary outcome.

## Materials and methods

We performed a multi-centre randomised controlled trial comparing ASC and LSC in four teaching and two university hospitals in the Netherlands. All hospitals take part in the Dutch consortium for women’s health. The consortium is a collaborative network in clinical studies in the field of obstetrics and gynaecology. The study was approved by the ethical committee of the Máxima Medical Centre in Veldhoven (file number NL12130.015.06) and the Board of Directors of all participating hospitals, and was registered in the Dutch Trial Register (NTR3276).

Eligible women with vault prolapse who met the inclusion criteria were counselled about the trial. Vault prolapse was defined as a post-hysterectomy prolapse of the apical compartment. After written informed consent was given, randomisation was performed by an independent research secretariat located in Amsterdam after a phone call or e-mail by the coordinating investigator. The treatment allocation was done by opaque sealed envelopes in a 1:1 ratio to either laparoscopic sacrocolpopexy or open abdominal sacrocolpopexy. Women received a randomised case number to ensure that their data would be treated anonymously. No changes were made to the protocol after trial commencement, other than including more participating centres.

We included women with a history of hysterectomy presenting with symptomatic vaginal vault prolapse, with or without concomitant cystocele and rectocele, who chose to undergo surgery. Women who had undergone previous surgical correction of a vault prolapse were excluded, in addition to women with a contra-indication for a surgical intervention because of their general physical condition.

### Surgical intervention

The intervention was either abdominal or laparoscopic sacrocolpopexy following randomisation. To exclude a learning curve for both surgical interventions and procedure bias, all participating gynaecologists had to have performed at least 50 procedures before the start of the study. The procedures were standardized as much as possible to confirm consistency. Participants received a bowel preparation the day before the operation. Prophylactic antibiotics were given peroperatively (metronidazole/cefazolin). As prophylaxis for thromboembolism per- and postoperatively subcutaneous low molecular weight heparin was administered.

#### Abdominal sacrocolpopexy

The abdominal sacrocolpopexy was performed by a laparotomy under general anaesthesia, preferably using a Pfannenstiel incision. The peritoneum from the promontory to the vault was incised to expose the rectovaginal and vesicovaginal fascia, extending to the sacral promontory. A type 1 polypropylene mesh was used, which was cut into two pieces 3 cm wide and approximately 15 cm long. One piece of the mesh was attached between the vagina and the bladder anteriorly, and another as far down the posterior vaginal wall as possible using Ethibond, non-absorbable, synthetic and multifilament sutures from Ethicon. The mesh was fixated to the anterior part of the vaginal vault with four stitches, and six stitches were used to fixate the mesh posterior. The two meshes were sutured to each other, after which only the posterior mesh was fixed to the longitudinal vertebral ligament by staples or non-absorbable sutures, depending on surgeon preference. Excess mesh was trimmed and removed. The mesh was re-peritonealised.

#### Laparoscopic sacrocolpopexy

Laparoscopic sacrocolpopexy was performed under general anaesthesia with four trocars, one for the scope and three side trocars. The essence of the procedure was the same as for the abdominal procedure. The vaginal vault was elevated with a vaginal probe. The peritoneum from the promontory to the vault was incised laparoscopically by scissors to expose the rectovaginal and vesicovaginal fascia. One piece of type 1 polypropylene mesh was attached anteriorly and another as low as possible on the posterior vaginal wall. The sutures, size of the mesh and its fixation were the same as in the abdominal procedure. The mesh was attached to the sacral promontory using staples and was peritonealised. All centres used polypropylene meshes and the same sutures.

### Peroperative assessment

When stress incontinence was diagnosed preoperatively, it was up to the patient and her gynaecologist whether incontinence surgery was performed during the same procedure or in a second operation after evaluation of the sacrocolpopexy on the stress incontinence. A tension-free vaginal tape was used if incontinence surgery was indicated. No Burch colposuspensions were performed. Both procedures could be completed with any necessary concomitant vaginal operation after the vault suspension has been carried out. The decision to perform additional prolapse surgery was made by the surgeon after the sacrocolpopexy was completed.

A urethral catheter was left in situ and was removed at the first day postoperatively or as clinically indicated. If the procedure was complicated by a bladder lesion, the catheter was removed after 1 week. In the case of urinary retention after removal of the catheter on the first day, the catheter was re-inserted for another day.

### Outcome measures

Women were sent a questionnaire preoperatively, at 3–6 months postoperatively and 12 months postoperatively. Women were asked to undergo a pelvic examination preoperatively and at 6 weeks and 12 months postoperatively. The observer was an independent researcher/resident, who had not performed the surgery. The researcher was not blinded to the type of surgery.

The primary outcome of the study was functional outcome, which was evaluated using the Urinary Distress Inventory (UDI) at 12 months’ follow-up [18]. The UDI is a validated questionnaire evaluating prolapse-related symptoms. The questionnaires also contain versions of the Defecatory Distress Inventory (DDI) [19], the Incontinence Impact Questionnaire (IIQ) [18] and the Patient Global Impression of Improvement (PGI-I) [20] and questions about sexuality, which were secondary outcomes. Other secondary outcomes were procedure time, amount of estimated blood loss and hospital stay, perioperative complications, re-interventions and long-term complications. Re-intervention included incontinence or prolapse surgery. All collected data were registered in a case report form. Another secondary outcome was the composite outcome of success, defined as no prolapse beyond the hymen, no bothersome bulge symptoms, and no repeat surgery or pessary use for recurrent prolapse within 12 months [20, 21]. Remaining study parameters were body mass index, pre- or postmenopausal status, use of oestrogens, combined prolapse surgery or stress urinary incontinence procedures. The anatomical outcome using the Pelvic Organ Prolapse Quantification system (POP-Q) [22] was the secondary end-point. A pelvic examination was performed to evaluate the anatomical results of the prolapse repair.

### Sample size

A difference between the two surgical techniques of 10 points between the two groups on the prolapse domain of the UDI 12 months after surgery was considered to be clinically relevant. Assuming a standard deviation of the score on this domain of 15 points, we needed 74 participants to show a statistically significant difference in the primary outcome (power of 80%, α error 0.05) [23].

### Statistical analysis

The trial was a prospective, randomised controlled trial conducted with the aim of determining the superiority of the primary endpoint (prolapse domain of the UDI) in the laparoscopic sacrocolpopexy group. Analysis was by intention to treat. The domain scores were calculated for the UDI, DDI and IIQ. To examine differences between groups we used an unpaired *t* test or Mann–Whitney test for continuous variables depending on the distribution, whereas a Chi-squared test was used for dichotomous variables. We used two-sided significance tests, and a *p* value <0.05 was considered to indicate statistical significance. For dichotomous outcomes, we calculated relative risks and 95% confidence intervals. We used the statistics package SPSS version 22 (IBM, Armonk, NY, USA).

## Results

**Fig. 1:**
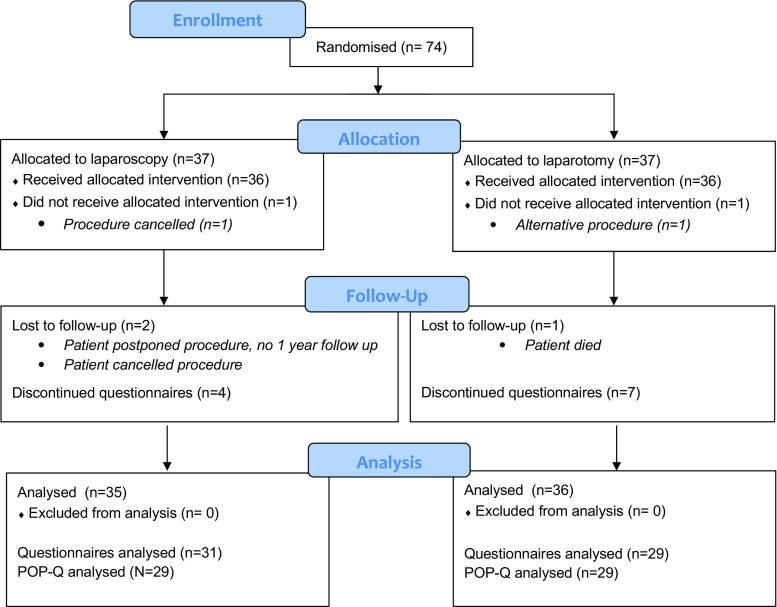
Patient flow through each stage of the study

The results are reported by means of the IUGA/ICS recommendations for reporting outcomes of surgical procedures for pelvic organ prolapse [24] and the CONSORT statement (www.consort-statement.org). Between 2007 and 2012, we randomised 37 women to the laparoscopic sacrocolpopexy group and 37 to the open group (Fig. 1). One woman randomised to the laparoscopy group was very satisfied with a pessary, which she received to cover the time until the operation, and she cancelled surgery. In the abdominal group, one patient underwent a sacrospinous fixation of the vault prolapse because she was not happy with the randomisation result. Both women were included in the intention-to-treat analysis. In the laparoscopic group one procedure was combined with concomitant vaginal surgery, versus 3 in the open group. In both groups one procedure was combined with a tension-free vaginal tape (TVT-O). In the laparoscopic group no concomitant vaginal prolapse surgery was performed, whereas in the open group, two procedures were combined with a posterior colporrhaphy.

At 12 months’ follow-up, there were 14 questionnaires missing, of which 11 participants (15.5%) were unwilling to complete the questionnaires, 1 participant did not receive the intervention, 1 participant postponed the procedure until the end of the study period for private reasons and had not yet completed the 1-year follow-up, and 1 patient died 5 days after the intervention because of a complication of the intervention. The number of missing questionnaires is presented in Fig. 1. All non-responders were contacted by telephone and most of them explained that they were doing well, which was a reason not to return the questionnaires. Patient characteristics of responders and non-responders were comparable.

Table 1 shows the baseline characteristics of the study population. The median age of the study population was 65.2 (IQR 61–71) years in the laparoscopic group and 66.7 (IQR 64–73) in the abdominal group. Other baseline characteristics were also comparable, including the preoperative POP-Q stage

**Table 1:**
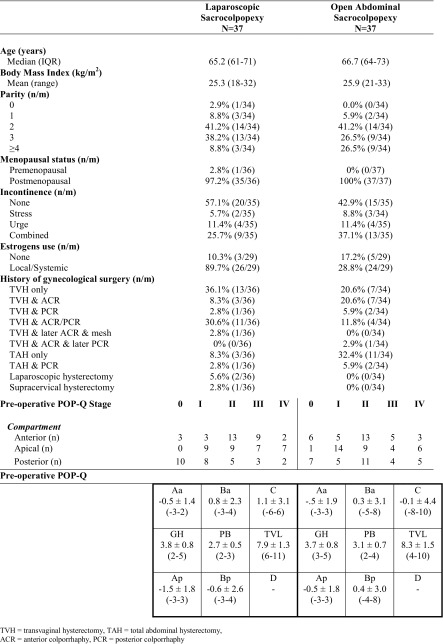
Baseline characteristics

Table 2 shows UDI, DDI and IIQ scores before surgery and 12 months after surgery. Both groups reported after 12 months a UDI score of 0.0 (IQR: 0–0) for the domain “genital prolapse” (*p* = .93), which was the primary outcome. The domain “pain and discomfort” showed a score of 0.0 (IQR: 0–29) for the laparoscopic group versus 8.3 (IQR: 0–33) for the abdominal group (*p* = 0.15). The UDI domain scores improved significantly for both groups at 12 months post-surgery (*p* ≤ 0.001). At 12 months’ follow-up, the PGI-I score of “very much better” was 25% (8 out of 31) for the laparoscopy group, and 26% (7 out of 27) for the open abdominal group. If we add the score of “much better” the percentages are 71% (22 out of 31) and 74% (20 out of 27), which was not statistically different (*p* = 0.563).

**Table 2:** Domain scores for disease-specific quality of life

	Pre-operative		12 months post-operative		*p-value*
	Laparoscopic	Abdominal	Laparoscopic	Abdominal	
	N = 34	N = 31	N = 31	N = 29	
Urogenital distress inventory					
Overactive bladder					
Median (IQR)	33.3 (11–56)	44.4 (22–50)	0.0 (0–11)	5.6 (0–19)	*.30*
Incontinence					
Median (IQR)	16.7 (0–50)	16.7 (0–42)	16.7 (0–33)	16.7 (0–33)	*.52*
Obstructive micturition					
Median (IQR)	0.0 (0–33)	16.7 (0–58)	0.0 (0–13)	0.0 (0–0)	*.28*
Pain/Discomfort					
Median (IQR)	16.7 (0–50)	33.3 (17–33)	0.0 (0–29)	8.3 (0–33)	*.15*
Genital prolapse					
Median (IQR)	66.7 (58–92)	66.7 (33–67)	0.0 (0–0)	0.0 (0–0)	*.93*
Recurrent bladder infections (n/m)					
Never	22 (65%)	17 (57%)	26 (84%)	21 (75%)	.50
Once	8 (24%)	4 (13%)	4 (13%)	4 (14%)	
Between 2–4 times	1 (3%)	5 (17%)	0 (0%)	2 (7%)	
More than 4 times	3 (9%)	4 (13%)	1 (3%)	1 (4%)	
Incontinence de novo					
Urge incontinence			2	3	*.23*
Stress incontinence			5	4	*.69*
Defecatory distress inventory					
Constipation					
Median (IQR)	0.0 (0–17)	0.0 (0–33.3)	0.0 (0–17)	0.0 (0–17)	*.76*
Obstructive defecation					
Median (IQR)	4.2 (0–17)	8.3 (0–25)	0.0 (0–8)	0.0 (0–8)	*.56*
Pain/Discomfort					
Median (IQR)	0.0 (0–0)	0.0 (0–0)	0.0 (0–0)	0.0 (0–17)	*.03*
Incontinence					
Median (IQR)	0.0 (0–17)	8.3 (0–33)	0.0 (0–0)	0.0 (0–17)	*.13*
Incontinence flatus					
Median (IQR)	33.3 (0–67)	33.3 (0–67)	0.0 (0–33)	0.0 (0–17)	*.48*
Incontinence impact questionnaire					
Physical					
Median (IQR)	25.0 (0–50)	0.0 (0–33)	0.0 (0–25)	0.0 (0–17)	*.66*
Mobility					
Median (IQR)	11.1 (0–33)	33.3 (11–44)	0.0 (0–28)	11.1 (0–25)	*.37*
Social					
Median (IQR)	11.1 (0–22)	11.1 (0–33)	0.0 (0–6)	0.0 (0–11)	*.47*
Shame					
Median (IQR)	0.0 (0–17)	16.7 (0–17)	0.0 (0–8)	0.0 (0–17)	*.92*
Emotional					
Median (IQR)	11.1 (0–33)	22.2 (0–33)	0.0 (00–22)	0.0 (0–25)	*.54*
Sexuality					
Sexually active	20 (63%)	14 (45%)	26 (93%)	26 (93%)	1.00
Dyspareunia					.23
Bother:					
Not at all	11	5	14	10	
Moderately	0	3	3	3	
Somewhat	4	4	1	0	
Quite a bit	2	1	0	0	
Not applicable	14	18	8	15	
Frequency coitus					.66
Never	17	18	11	15	
<1×/month	4	5	3	4	
1-2×/month	4	3	9	6	
1×/week	6	3	4	1	
>1×/week	1	2	1	2	

Clinical outcomes are presented in Table 3. In the laparoscopic sacrocolpopexy group blood loss was 86 mL (IQR 10–100) vs 200 mL (IQR 100–300) in the abdominal group (*p* < 0.001). Hospital stay was 2 days (IQR 2–3) vs 4 days (IQR 3–5; *p* < 0.001). Duration of surgery (125 vs 115 min; *p* = 0.31), number of complications during surgery (5.6% vs 0%, *p* = 0.15), and number of complications during admission (5.6% vs 18.9%, *p* = 0.06) were not statistically significant different.

**Table 3:** Clinical outcome

	Laparoscopic sacrocolpopexy	Open abdominal sacrocolpopexy	*p-value*
	N = 36	N = 37	
Operative time (minutes)			
Median (IQR)	125 (108–135)	115 (94–129)	*.31*
Estimated blood loss (ml)			
Median (IQR)	86 (10–100)	200 (100–300)	*<.001*
Hospital stay (days)			
Median (IQR)	2 (2–3)	4 (3–5)	*<.001*
Complications during surgery (n/m)	5.6% (2/36)	0% (0/36)	*.15*
Bladder lesion (conversion)	1	0	
Bleeding (conversion)	1	0	
Complications during admission (n/m)	5.6% (2/36)	18.9% (7/37)	*.06*
Fatal bowel perforation	0	1	
Wound dehiscence	0	2	
Pulmonary embolism	0	1	
Ileus	0	3	
Wound infection	1	0	
Pyelonephritis (re-admission)	1	0	

The laparoscopic group contains fewer complications, 4 in the laparoscopic group versus 7 in the open group, which is not significantly different (RR 0.57; 95% CI 0.50–2.27). In the open abdominal group the complications that occurred were more severe. One complication concerned a 79-year-old woman who presented with cardiac arrhythmia on the third day after surgery. She was diagnosed with sepsis and a bowel perforation was suspected. A relaparotomy was performed and the diagnose bowel perforation could be confirmed. She developed pneumonia and due to multi-organ failure, she died on the fifth day after surgery. The complication was considered a calamity and reported to the health care inspectorate.

Two other women in the open abdominal group had wound dehiscence that needed to be repaired surgically. One procedure carried out in the laparoscopic group had to be converted because of bleeding coming from the promontory. The total estimated blood loss of this procedure was 1,200 mL.

Table 4 shows the surgical re-interventions for pelvic organ prolapse and occult/new urinary incontinence. In the laparoscopy group, there were 7 women in whom a re-intervention for prolapse or incontinence was performed, versus 4 in the open surgery group (RR 1.75; 95% CI 0.81–3.91). In both groups, 3 women had surgery for stress urinary incontinence. The laparoscopic group had 4 re-interventions for recurrent POP versus 1 in the open group (RR 4, 95% CI 0.84–5.73). All surgical re-interventions concerned the posterior compartment. No pessaries were placed as a re-intervention. At 12 months’ follow-up, 2 participants in the laparoscopic group developed de novo urge incontinence, and 5 de novo stress incontinence, versus 3 and 4 respectively in the open abdominal group according to the questionnaires. There was no significant difference in these results between the groups.

**Table 4:** Surgical re-interventions for pelvic organ prolapse and occult/new urinary incontinence

	Laparoscopic sacrocolpopexy n = 36	Open abdominal sacrocolpopexy n = 37	*p value*
Re-intervention (n/m)	16.7% (7/36)	10.8% (4/37)	0.12
Incontinence surgery	3	3	1.00
TVT-S	1	0	
TVT-O	1	3	
TOT	1	0	
Prolapse surgery	4	1	0.17
Rectopexy	1	0	
Posterior colporrhaphy	2	0	
Enterocele repair	1	0	
Posterior vaginal mesh	0	1	

There were no significant differences between the groups in anatomical results 12 months postoperatively according to the POP-Q, as shown in Table 5. At the 12-months postoperative follow-up visit no mesh or suture exposure was seen during vaginal examination in the two groups. No other complications were seen at the 12 months’ follow-up visit.

**Table 5:**
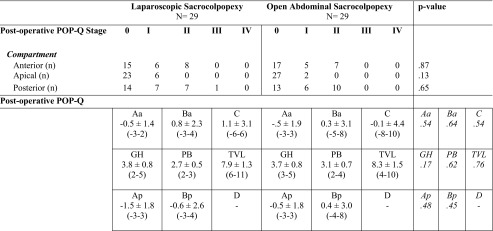
POP-Q 12 months postoperatively

We asked our population at the 12 months’ follow-up visit about their complaints and 4 of the participants mentioned (unexplained) pelvic pain; 1 in the laparoscopic group and 3 in the open abdominal group. In all four of these participants, pelvic pain was already present before the surgery, but it turned out to be worse 12 months after the procedure. If we look at the questionnaires, 8 participants in the laparoscopic group vs 13 in the abdominal group had pelvic pain after 12 months, which was not a significant difference (*p* = 0.056).

The composite outcome of success was 83.8% (31 out of 37) for the laparoscopy group and 89.2% (33 out of 37) in the open abdominal group. In both groups, there were no recurrences of stage 2 or higher of the apical compartment. Two patients in the laparoscopy group had bothersome bulge symptoms compared with 4 in abdominal group. Five participants of the laparoscopy group were re-operated for POP, versus 1 in the abdominal group.

According to the questionnaires, in both groups more participants became sexual active, there was less dyspareunia and the coitus frequency was increased at 12 months postoperatively (Table 2). There are no significant differences between the groups.

## Discussion

### Main findings

We performed a multicentre randomised trial that compared laparoscopic and open abdominal sacrocolpopexy in patients with a vaginal vault prolapse. There were no significant differences in quality of life related to micturition, prolapse and defecation in the two groups. Anatomical results were similar at 12 months after surgery. In the laparoscopic group, there was less blood loss during the procedure and the hospital stay was shorter.

Quality of life was the primary outcome in our trial. In both groups, the functional outcomes of the UDI domain scores were significantly improved, which supports previous findings of a high success rate for sacrocolpopexy [3–5]. Disease-specific quality of life was statistically equal after both laparoscopic and open abdominal sacrocolpopexy. These results are in line with those of a randomised controlled trial by Freeman et al. comparing open abdominal with laparoscopic sacrocolpopexy, which was published recently [15]. In this study [15], there was also less blood loss and a shorter hospital stay after laparoscopy.

We chose disease-specific quality of life, using the UDI questionnaire, as the primary outcome of our study. As outcome definitions for evaluating prolapse surgery were improved after the start of the trial, after a publication by Barber et al. [25], we added the combined outcome measure (recurrent pelvic organ prolapse stage 2 or higher in the apical compartment, with bothersome bulge symptoms, and re-interventions), at 12 months’ follow-up. This outcome measure was not specified in the study protocol.

There was no significant difference in anatomical outcome between the two groups in this trial [15]. These results correspond to the outcomes of our study. The results of similar functional and anatomical effects, and less blood loss and shorter hospital stay were also found in two other comparative cohort studies [16, 17].

We showed that sacrocolpopexy is an effective surgical treatment for women with a symptomatic vault prolapse. Although the focus of the sacrocolpopexy is mainly the apical and the anterior compartment, the posterior compartment improves as well. Besides anatomical improvement, patients have better scores in all domains of the disease-specific quality of life questionnaires.

There was a trend towards fewer complications in the laparoscopic group (11% vs 18.9%, RR 0.57; 95% CI 0.50–2.27). The complications in the open group were much more severe, including re-laparotomies and a fatal bowel perforation.

The study by Freeman et al. did not show any significant differences in complication rates either: 5.6% (2 out of 26) in the laparoscopic vs 7.4% (2 out of 27) in the open group. Complications in the laparoscopic group included opening of the vagina and one bladder injury. In the open group an area of mesentery of the small bowel became detached and this required the resection of 10 cm of small bowel. In one other case, there was excessive bleeding from the sacrum, which required haemostatic bone wax [15].

One reason for our unexpected higher complication rate may be accurate documentation during a prospective trial. The trial consists of an unselected study population, in contrast with retrospective cohort studies. Furthermore, patients were referred from other centres for the sacrocolpopexy, which may influence the complexity of the patient population. Despite these possible explanations, it is still unusual that so many severe and rare complications occurred during this trial.

We did not see any mesh or suture exposure in our study population. Other trials reported rates of mesh-related complications of between 3 and 11% [5–8]. Our absence of mesh complications may be because our follow-up time was only 1 year, which is relatively short for the development of erosion.

The anatomical results of the initial surgery were similar, but participants who had undergone laparoscopic surgery had more re-interventions. The laparoscopic group had 4 re-interventions for recurrent POP versus 1 in the open group (RR 4, 95% CI 0.84–5.73), all concerning the posterior compartment. An explanation could be that two open procedures were combined with a posterior colporrhaphy in the same session versus no concomitant vaginal POP surgery in the laparoscopic group.

The inclusion period of our trial was 5 years, which is a long period for a multicentre trial with six participating centres. Many patients and gynaecologists preferred the laparoscopic procedure and the laparoscopic sacrocolpopexy was already being implemented in many participating centres, despite the fact that its clinical effectiveness was still unknown. Unfortunately, not all eligible patients were documented. Most participants were randomised in the last 3 years of the study by including more centres. Moreover, many procedures were performed by the same surgeon, as this gynaecologist visited some of the other sites to perform the laparoscopic sacrocolpopexy for the study population. The other procedures were performed by experienced surgeons who had been trained to perform the procedure the same way. This resulted in a homogeneous operation technique and frequent performance of the procedure.

### Strengths and limitations

We performed a randomised controlled trial, which is considered the best type of study to assess the effectiveness of a procedure. Another strength of our trial is that procedures were all performed by experienced gynaecologists with a track record of more than 50 sacrocolpopexy procedures. A trial of Deprest et al. showed that it takes 60 procedures to effectively limit complications, owing to the challenging suture and dissection skills that are needed for this technique [14]. The laparoscopic sacrocolpopexy is a challenging, level 4 procedure. The laparoscopic technique has an advantage over an open abdominal procedure with regard to dissection, which is easier during laparoscopy because of the increased visual field. However, stitching is more difficult compared with the open technique because of a decreased degree of movement and two-dimensional vision. As a large number of patients are needed to acquire sufficient surgical skills, this procedure should only be performed by experienced surgeons.

A limitation of our study was the relatively high percentage of loss to follow-up (15.5%). The number of missing questionnaires was equal in the two groups. All non-responders were contacted by telephone and most of them explained that they were doing well, which was a reason not to return the questionnaires. However, patient characteristics of responders and non-responders were comparable; thus, we do not believe that the loss to follow-up has greatly affected our results.

Another limitation is that the patients and staff were not blinded to the intervention. Although patients were encouraged by the medical care staff to recover quickly and to not extend their admission for unnecessary reasons, there is still a chance of bias because of the type of incision that was used. This could affect the length of the hospital stay; however, 2 vs 4 days still constitutes a large difference of 2 days. Furthermore, the hospital stay was prolonged by the extended (re)admission because of several complications in the abdominal group.

### Interpretation

In conclusion, this randomised controlled trial comparing laparoscopic and open abdominal sacrocolpopexy showed no significant differences in functional and anatomical outcome, but there was less blood loss and a shorter hospital stay was shorter if the procedure was performed using the laparoscopic approach. Although this superiority study did not show a significant difference in the primary outcome (UDI prolapse domain), there is still evidence to support a laparoscopic approach as there was less blood loss, the hospital stay was shorter, and the anatomical and combined outcomes were not statistically different. Therefore, we recommend further implementation of the laparoscopic approach. However, given the learning curve, we advise low-volume centres to refer patients to a centre with laparoscopic expertise.

## Conclusion

Our trial provides evidence to support a laparoscopic approach when performing sacrocolpopexy, as there was less blood loss and the hospital stay was shorter, whereas functional and anatomical outcomes were not statistically different.

## References

[1] Dällenbach P, Kaelin-Gambirasio I, Jacob S, Dubuisson JB, Boulvain M (2008). Incidence rate and risk factors for vaginal vault prolapse repair after hysterectomy. Int Urogynecol J Pelvic Floor Dysfunct.

[2] Mant J, Painter R, Vessey M (1997). Epidemiology of genital prolapse: observations from the Oxford family planning association study. BJOG.

[3] Maher C, Feiner B, Baessler K, Schmid C (2013). Surgical management of pelvic organ prolapse in women (review). Cochrane Database Syst Rev.

[4] Snyder TE, Krantz KE (1991). Abdominal-retroperitoneal sacral colpopexy for the correction of vaginal prolapse. Obstet Gynecol.

[5] Timmons MC, Addison WA, Addison SB, Cavenar MG (1992). Abdominal sacral colpopexy in 163 women with posthysterectomy vaginal vault prolapse and enterocele: evolution of operative techniques. J Reprod Med Obstet Gynecol.

[6] Nygaard I, Brubaker L, Zyczynski HM, Cundiff G, Richter H, Gantz M (2013). Long-term outcomes following abdominal sacrocolpopexy for pelvic organ prolapse. JAMA.

[7] Ganatra AM, Rozet F, Sanchez-Salas R, Barret E, Galiano M, Cathelineau X (2009). The current status of laparoscopic sacrocolpopexy: a review. Eur Urol.

[8] Hilger WS, Poulson M, Norton PA (2003). Long-term results of abdominal sacrocolpopexy. Am J Obstet Gynecol.

[9] Nygaard IE, McCreery R, Brubaker L, Connolly A, Cundiff G, Weber AM (2004). Abdominal sacrocolpopexy: a comprehensive review. Obstet Gynecol.

[10] Parkes IL, Sveiky D (2014). Sacrocolpopexy for the treatment of vaginal apical prolapse: evidence based surgery. J Minim Invasive Gynecol.

[11] Khan A, Alperin M, Wu N, Clemens WQ, Dubina E, Pashos CL (2012). Comparative outcomes of open versus laparoscopic sacrocolpopexy among medicare beneficiaries. Int Urogynecol J.

[12] Nezhat CH, Nezhat F, Nezhat C (1994). Laparoscopic sacral colpopexy for vaginal vault prolapse. Obstet Gynecol.

[13] Claerhout F, Deprest J (2014). Analysis of the learning process for laparoscopic sacrocolpopexy: identification of challenging steps. Int Urogynecol J.

[14] Deprest J, Roovers JP (2014). The challenge of implementing laparoscopic sacrocolpopexy. Int Urogynecol J.

[15] Freeman RM, Pantazis K, Thomson A, Frappell J, Bombieri L, Moran P (2012). A randomised controlled trial of abdominal versus laparoscopic sacrocolpopexy for the treatment of post-hysterectomy vaginal vault prolapse: LAS study. Int Urogynecol J.

[16] Paraiso MF, Walters MD, Rackley RR, Melek S, Hugney C (2005). Laparoscopic and abdominal sacral colpopexies: a comparative cohort study. Am J Obstet Gynecol.

[17] Klauschie JL, Suozzi BA, O’Brien MM, McBride AW (2009). A comparison of laparoscopic and abdominal sacral colpopexy: objective outcome and perioperative differences. Int Urogynecol J Pelvic Floor Dysfunct.

[18] Van der Vaart CH, de Leeuw JR, Roovers JP, Heintz AP (2003). Measuring health-related quality of life in women with urogenital dysfunction: the urogenital distress inventory and incontinence impact questionnaire revisited. Neurourol Urodyn.

[19] Roovers JP, van der Blom JG, van der Vaart CH (2008). Hysterectomy does not cause constipation. Dis Colon Rectum.

[20] Srikrishna S, Robinson D (2010). Validation of the patient global impression of improvement (PGI-I) for urogenital prolapse. Int Urogynecol J Pelvic Floor Dysfunct.

[21] Chmielewski L, Walters MD (2011). Reanalysis of a randomized trial of 3 techniques of anterior colporrhaphy using clinically relevant definitions of success. Am J Obstet Gynecol.

[22] Bump RC, Mattiasson A, Bø K, Brubaker LP, DeLancey JO, Klarskov P (1996). The standardization of terminology of female pelvic organ prolapse and pelvic floor dysfunction. Am J Obstet Gynecol.

[23] Roovers JP, van der Vaart CH, van der Bom JG, van Leeuwen JH, Scholten PC, Heintz AP (2004). A randomised controlled trial comparing abdominal and vaginal prolapse surgery: effects on urogenital function. Br J Obstet Gynaecol.

[24] Toozs-Hobson P, Freeman R (2012). An international urogynaecological association (IUGA)/international continence society (ICS) joint report on the terminology for reporting outcomes of surgical procedures for pelvic organ prolapse. Int Urogynecol J Pelvic Floor.

[25] Barber MD, Burbaker L, Nygaard I, Wheeler TL, Schaffer J, Chen Z (2009). Defining success after surgery for pelvic organ prolapse. Obstet Gynecol.

